# A Sampling-Based Approach for Achieving Desired Patterns of Probabilistic Coverage with Distributed Sensor Networks

**DOI:** 10.3390/s23135999

**Published:** 2023-06-28

**Authors:** Russell Costa, Thomas A. Wettergren

**Affiliations:** Naval Undersea Warfare Center, 1176 Howell Street, Newport, RI 02841, USA; russell.costa.civ@us.navy.mil

**Keywords:** distributed sensor networks, coverage, placement, optimization, sampling

## Abstract

A new method is derived for finding the best positions in which to locate the sensors in a distributed sensor network in order to achieve a desired variation, or pattern, in spatial coverage over a specified domain. Such patterning is important in situations when there are not enough sensors to completely cover a region adequately. By providing coverage based on a desired pattern, this approach allows a user/designer to specify which sub-regions of the domain are more important to cover, and to what level that is desired. The method that is developed is novel in that it is an analytic approach, as opposed to existing numerical optimization approaches, and thus provides solutions rapidly and can also be applied to provide online repositioning for existing sensor networks to respond to changes in the environment. The method is based on deriving an expression for the probabilistic density of sensor locations that best matches the desired coverage under given spatially varying environmental conditions; and then samples from that sensor density to determine specific sensor locations. The performance of the method is demonstrated on numerical examples in both one-dimensional and two-dimensional settings. Comparisons are made between solutions found from this approach and solutions obtained by a numerical optimization technique.

## 1. Introduction

The quality with which a distributed sensor network can cover a region is heavily dependent on the relative locations of the sensors. This pattern of sensor placement becomes even more critical when the number of available sensors is limited due to either cost or availability concerns. For most initial planning instances, there are available computing resources to run large optimization computations to determine the best pattern to employ. As these optimal patterns depend on the local sensor performance characteristics, which themselves often depend on the location of the individual sensors, the optimal configuration can often change as environmental characteristics change. It can also change as the composition of available sensors changes (due to additions and/or deletions from the network). Due to these performance changes, replanning of the locations of sensors is often required, and in such situations the large computing resources available for initial planning may not be as readily accessible. Thus, having robust methods to determine an optimal configuration rapidly becomes a more pressing issue with regard to maintaining a distributed sensor network over an extended period of time.

The development of advanced wireless communications, smaller microelectronics, extended battery capacity, and improved manufacturing techniques that occurred near the end of the last century made distributed sensor networks a practical reality. These systems have been used in a variety of applications to provide a remote capability for monitoring regions of interest. As pointed out in multiple early surveys on the topic [1,2], the determination of a good set of locations for the sensors is an important aspect to the system design problem. In many cases, the consideration of interest in choosing sensor placements is to maintain the coverage capability of the overall distributed sensor network [3]. In homogeneous domains, obtaining a certain quality (or level) of coverage is akin to the covering problem for facility location planning from operations research [4]. Such problems involve the requirement to find the proper amount of overlap between locations, and are therefore sometimes referred to as the related operations research problem of cooperative covering [5,6]. In order for a sensor network to be practical, these coverage goals must be balanced against other considerations such as the connectivity [7], the quality of service [8], or the operational serviceability of the system [9].

Generally, the approaches to determining the optimal locations of sensors to provide coverage involve writing a version of the coverage performance objective and then performing a numerical optimization procedure (or algorithm that approximates optimization) to find the positions. Deif and Gadallah [10] have taken the variety of approaches and categorized them into four principal categories: genetic algorithms, computational geometry, artificial potential fields, and particle swarm optimization. Some other approaches apply simulated annealing techniques [11] or matheuristics that combine genetic algorithms with integer linear programming [12]. Many of the most utilized approaches are grid-based, as they involve the selection of a limited number of points from a set of potential locations on a grid. This has been used successfully for standard coverage [13] as well as for coverage with other considerations [14]. The computational geometry approaches often revolve around efficient techniques for finding various partitions of the space, such as Voronoi diagrams [15]. Many of the most commonly used techniques for placing distributed sensors for coverage rely on genetic algorithms, as they have become common when solving the related facility location problems in operations research [16]. These genetic algorithm approaches either work directly with the sensor positions [17] or use some alternative representation of a group of sensor positions such as density functions [18].

While most distributed sensor network problems are stated as two-dimensional applications, there are also three-dimensional applications as well as some very practical one-dimensional distributed sensor networks. The most common one-dimensional applications are where a distributed sensor network is used as a barrier for entry in a region [19,20]. Similar to the planar problems, determining the placements for sensors in these one-dimensional barrier-style problems can also involve additional considerations such as maintaining energy efficiency [21] and maximizing network lifetime [22]. To maintain performance along the line, regular motion of the sensors in order to reconfigure the locations is a desired feature. Such motion patterns have been developed as moving from the interior of a domain to the barrier [23], as a control problem along the line [24], and as a self-organizing principle for independent agents [25]. These various motion approaches all revolve around the same goal of reconfiguration to handle changes in either the sensors or the environment. Efficient computation of sensor locations that maintain the desired coverage characteristics is an important step to achieving such goals.

Regardless of the dimensionality of a distributed sensor network application, the selection of the placement of sensors is an important component to the system design. Complexities such as a non-homogeneous environment and/or a non-uniform coverage goal make the placement problem more complicated than a standard geometric packing problem. While many approaches are available for numerically solving such a problem as an optimization routine, they are computationally intensive processes. In situations in which the distributed sensor network is to be either rapidly deployed or adapted once already deployed, it becomes important to find procedures for determining the proper placement of sensors rapidly. As such, in this paper an analytic approach is presented for making such placement decisions that is based on sampling from a desired sensor distribution. In the next section, an analytic approximation is formulated for the desired sensor distribution and then shown how it can be sampled through a deterministic sampling process. The following section shows numerical examples of the approach and compares them to solutions that are obtained through a genetic algorithm optimization process. The genetic algorithm is used to achieve a detailed numerical solution for the achievable match that can be made in a given example; this provides a baseline of the achievable match that can be obtained when computational effort is not an issue. The examples presented include both one-dimensional and two-dimensional scenarios.

## 2. Determining Sensor Positions

Define a pattern of probabilistic coverage as a function $\phi \left(x\right):R^{n}\to \left[0,1\right]$ that describes the level of coverage of various locations x∈Ω in the domain $\Omega \subset R^{n}$. In this context, the term coverage refers to the likelihood that an object located at a position *x* can be observed by the sensor network. This coverage provides a spatially varying measure of how well a sensor network can observe objects of interest. As ϕ(x) is spatially varying, it allows consideration of situations where some parts of the domain Ω are covered more effectively than others. The ability to create system configurations with desired patterns of nonuniform coverage levels is particularly important in situations where there are not enough sensors to provide idealized performance across the entire domain Ω; specifically it allows a designer to specify the relative importance of different portions of the domain Ω. The model assumes a desired pattern of probabilistic coverage ϕ(x) is given over the entire domain of interest (i.e., ϕ(x) is prescribed for all x∈Ω). The goal for selecting desired sensor positions is to find the set of positions that correspond to a sensor network whose resulting probabilistic coverage best matches the desired coverage ϕ(x). Hence, all of the desired performance characteristics are assumed to be subsumed in the coverage function ϕ(x).

### 2.1. Optimization Approach to Matching a Desired Coverage Pattern

Assume each location x∈Ω has associated with it a range $r\left(x\right):\Omega \to R^{+}$ and a detection probability $p_{d}\left(x\right):\Omega \to \left[0,1\right]$, such that a sensor placed at location *x* can observe objects that are located within a ball of radius r(x) that is centered around the sensor with probability $p_{d}\left(x\right)$. The coverage that is achieved by a set of *N* sensors located at positions $\left\{x_{i}\right\}$ is determined by first considering the coverage of each sensor as

(1) $$\begin{array}{c} \eta_{i}\left(x;x_{i}\right)=\left\{\begin{array}{cc} p_{d}\left(x_{i}\right), & |x-x_{i}|\leq r\left(x_{i}\right)\\ 0, & \mathrm{otherwise} \end{array}\right. \end{array}$$

Then the aggregate coverage of the group of *N* sensors is a probabilistic combination of the form

(2) $$\begin{array}{c} \eta \left(x;\left\{x_{i}\right\}\right)=1-\prod_{i=1}^{N}\left(1-\eta_{i}\left(x;x_{i}\right)\right) \end{array}$$

To best “match” a desired coverage level ϕ(x), an appropriate distance norm between ϕ(x) and $\eta (x;\left\{x_{i}\right\})$ is formed, and then the values of $\left\{x_{i}\right\}$ that minimize that distance are numerically computed. In particular, the Euclidean ($L^{2}$) norm is used to create the optimization problem:

(3) $$\begin{array}{c} \left\{x_{1}^{*},\ldots ,x_{N}^{*}\right\}=\underset{\left\{x_{1},\ldots ,x_{N}\right\}}{\mathrm{argmin}}\int_{\Omega }{\left(1-\phi \left(x\right)-\prod_{i=1}^{N}\left(1-\eta_{i}\left(x;x_{i}\right)\right)\right)}^{2}dx \end{array}$$

Direct optimization of Equation (3) is plausible, but computationally cumbersome. Thus, a new sampling procedure has been developed to find rapid solutions for a set of sensor positions that provide performance comparable to that which would be achieved by a full optimization approach. In the numerical results that follow, the numerically optimal solution is also computed for each example for comparison purposes.

### 2.2. Sampling Approach to Matching a Desired Coverage Pattern

For the direct optimization approach described above, the coverage function was formulated from the perspective of forming an expression for each sensor’s individual coverage, combining those expressions into an aggregate coverage expression, and then evaluating that combined expression at each location x∈Ω. This created an expression in Equation (2) that directly models the likelihood of covering each location x∈Ω, and the optimization approach used the difference between that likelihood and the desired coverage function as a numerical optimization objective (as shown in Equation (3)). For the sampling approach, an alternative perspective is taken in which the desired number of sensors at each location *x* is modeled based on the desired coverage and environmental conditions. That creates a density distribution of sensors that is then used in a deterministic sampling procedure to determine the desired locations for the specific sensors. The entire sampling-based approach is thus both analytic and deterministic, and therefore computationally rapid compared to the optimization approach.

Assume there are N(x) sensors that jointly cover some location x∈Ω (i.e., the regions corresponding to the individual coverage of the sensors that jointly overlap at *x*). This implies the probability of observing an object that is located at *x* is given by the local coverage function $\hat{\eta }\left(x;N\left(x\right)\right)$ according to

(4) $$\begin{array}{c} \hat{\eta }\left(x;N\left(x\right)\right)=1-{\left(1-p_{d}\left(x\right)\right)}^{N(x)} \end{array}$$

The number of sensors required to cover *x* to achieve a desired probabilistic quality of coverage ϕ(x) is then given by setting $\hat{\eta }\left(x;N\left(x\right)\right)=\phi \left(x\right)$ and solving for N(x) to obtain

(5) $$\begin{array}{c} N^{*}\left(x\right)=\frac{\log(1-\phi (x))}{\log(1-p_{d}\left(x\right))} \end{array}$$

where $N^{*}\left(x\right)$ is the desired number of sensors covering location *x*, and log(·) is a logarithm with any logarithm base (any logarithm base is allowed as long as both logarithms use the same base).

Assume that r(x) is smoothly varying, such that the set of sensor positions $x_{s}$ that cover location *x* is approximately the same as the set of sensors that are found within the ball of size r(x) around location *x*. Note that this smoothness assumption implies that r(x) almost always covers location $x_{s}$ when $r(x_{s})$ covers location *x* (and conversely). Let $r_{0}$ be the minimal detection range within the domain, such that

(6) $$\begin{array}{c} r_{0}=\min_{x\in \Omega }r\left(x\right) \end{array}$$

Now, for a situation where there are N(x) sensors in a ball of size r(x) around location *x*, then for sensors that are uniformly distributed within the ball, there are

(7) $$\begin{array}{c} N_{0}\left(x\right)=N\left(x\right)\;{\left(\frac{r_{0}}{r(x)}\right)}^{n} \end{array}$$

sensors uniformly distributed in a ball of size $r_{0}$ around location *x* (where *n* is the dimension of the space). Combining Equation (5) with Equation (7), it is seen that the number of desired sensors in a nominal ball of size $r_{0}$ around *x* is given by

(8) $$\begin{array}{c} N_{0}^{*}\left(x\right)=\left[\frac{\log(1-\phi (x))}{\log(1-p_{d}\left(x\right))}\right]\;{\left(\frac{r_{0}}{r(x)}\right)}^{n} \end{array}$$

The desired density distribution of sensors ρ(x) is given by considering the number of sensors $N_{0}^{*}\left(x\right)$ that are desired at each location *x* as well as the total number of available sensors $N^{TOT}$, yielding

(9) $$\begin{array}{c} N^{TOT}\rho \left(x\right)=\frac{N_{0}^{*}\left(x\right)}{|B(r_{0})|} \end{array}$$

where $|B(r_{0})|$ is the size of the ball of radius $r_{0}$ (where “size” corresponds to length in 1-D, area in 2-D, and volume in 3-D). Note that Equation (9) integrates to $N^{TOT}=N^{TOT}$ when integrated over the entire domain Ω, as expected. Thus, assuming there are $N^{TOT}$ available sensors for a distributed sensor network with desired coverage of ϕ(x), the desired density distribution of sensors ρ(x) is found through the substitution of Equation (8) into Equation (9) to explicitly yield

(10) $$\begin{array}{c} \rho \left(x\right)=\left[\frac{\log(1-\phi (x))}{|B\left(r_{0}\right)|N^{TOT}\log\left(1-p_{d}\left(x\right)\right)}\right]\;{\left(\frac{r_{0}}{r(x)}\right)}^{n} \end{array}$$

Equation (10) provides an analytic expression for the sensor density that depends on the physical characteristics of the space as well as the desired performance. Given a desired coverage ϕ(x) and total number of available sensors $N^{TOT}$, the corresponding sensor density function ρ(x) can be found from Equation (10), and then standard sampling methods can be used to find where to position individual sensors to best approximate this distribution.

Note that Equation (9) (and hence Equation (10)) implies that the total number of sensors $N^{TOT}$ exactly matches the number of sensors required to achieve the desired probabilistic quality. When there is that exact match, then the sensor density function ρ(x) in Equation (10) represents a proper probability density function. When there are more (or less) sensors than required, then the density function ρ(x) in Equation (10) is not a probability density function, but still represents the desired sensor density distribution, that is, the relative proportion of sensors that are desired in different portions of the domain Ω. Hence, in order to use the density function ρ(x) from equation in a sampling setting, form the sampling distribution f(x) from ρ(x) according to the standard scaling

(11) $$\begin{array}{c} f\left(x\right)=\frac{\rho (x)}{\int_{\Omega }\rho \left(x\right)\;dx} \end{array}$$

such that f(x):Ω→[0,1] and $\int_{\Omega }f\left(x\right)\;dx=1$, and thus f(x) can be utilized as a probability density function. The sampling approach that is employed is based on a deterministic sampling as opposed to stochastic sampling, as the large number of samples required to achieve the stochastic convergence are not expected. That is, stochastic sampling provides solutions that are asymptotically representative of the desired sampling distribution, but they require large numbers of samples to converge; whereas deterministic sampling provides reasonable estimates that are based on the number of samples that are taken. The sampling goal is to sample $N^{TOT}$ times according to the probability density function given by the sampling distribution f(x) from Equation (11) in order to have a set of $N^{TOT}$ sensor locations $\left\{x_{i}\right\}$ that are representative of the desired sensor density function ρ(x) from Equation (10). The deterministic sampling procedure that is employed forms the cumulative density function (CDF) F(x) from the probability density function f(x). As F(x) is a mapping from Ω→[0,1], $N^{TOT}$ points are sampled uniformly in the range [0,1] and then the corresponding points in Ω are determined according to the inverse mapping $F^{-1}$. For dimensions n>1, the mapping is not necessarily one-to-one, in which case solutions are randomly chosen from the solutions that meet the inverse criteria. These resulting $N^{TOT}$ sampled points x∈Ω represent the desired sensor locations from the sampling procedure. This sampling-based placement procedure is summarized in the pseudo-code shown as Algorithm 1.
**Algorithm 1** Compute Sensor Positions.**Input:** $N^{TOT},r\left(x\right),p_{d}\left(x\right)$ (sensor parameters)**Input:** ϕ(x) (desired coverage)**Output:** $\left\{x_{i}\right\}$ (sensor positions) /* Determine the minimum value of r(x) */$r_{0}\leftarrow r\left(0\right)$**for all** x∈Ω **do** **if** $r\left(x\right)<r_{0}$ **then**     $r_{0}\leftarrow r\left(x\right)$ **end if****end for** /* Compute the desired sensor density */$B_{0}\leftarrow$ size of ball of radius $r_{0}$ in dimension *n***for all** x∈Ω **do** $r^{ratio}\left(x\right)\leftarrow {\left(r_{0}\left(x\right)/r\left(x\right)\right)}^{n}$ $\rho \left(x\right)\leftarrow r^{ratio}\left(x\right)\left[\log\left(1-\phi \left(x\right)\right)/(B_{0}N^{TOT}\log\left(1-p_{d}\left(x\right)\right))\right]$**end for** /* Normalize density to a pdf and compute the cdf */**for all** x∈Ω **do** $f\left(x\right)\leftarrow \rho \left(x\right)/\int_{\Omega }\rho \left(x\right)\;dx$**end for**F(x)←CDF{f(x)} /* Sample the points uniformly from the cdf */**for** $i=1,\ldots ,N^{TOT}$ **do** $L_{i}\leftarrow \left(i-0.5\right)/N^{TOT}$ $x_{i}\leftarrow F^{-1}\left(L_{i}\right)$ **return** $x_{i}$**end for**

## 3. Numerical Examples

### 3.1. Example: Sensing along the Line

Consider a one-dimensional problem where sensors are to be placed along a line in a domain $\Omega \subset R^{1}$. Specifically, take Ω to be the interval 0≤x≤10. This line segment may represent a barrier in some surveillance application, or it may represent a region along which practical restrictions require all sensors to be on the same line. For whatever the practical application, for sensor placement purposes the only concern is that the sensors are to be placed in Ω to best achieve a desired level of probabilistic coverage ϕ(x).

For a first example, $N^{TOT}=8$ sensors are to be placed in Ω to meet a desired coverage of

(12) $$\begin{array}{c} \phi \left(x\right)=\left\{\begin{array}{cc} 0.5, & 0\leq x<5\\ 0.9, & 5\leq x\leq 8\\ 0.5, & 8<x\leq 10 \end{array}\right. \end{array}$$

**Figure 1:**
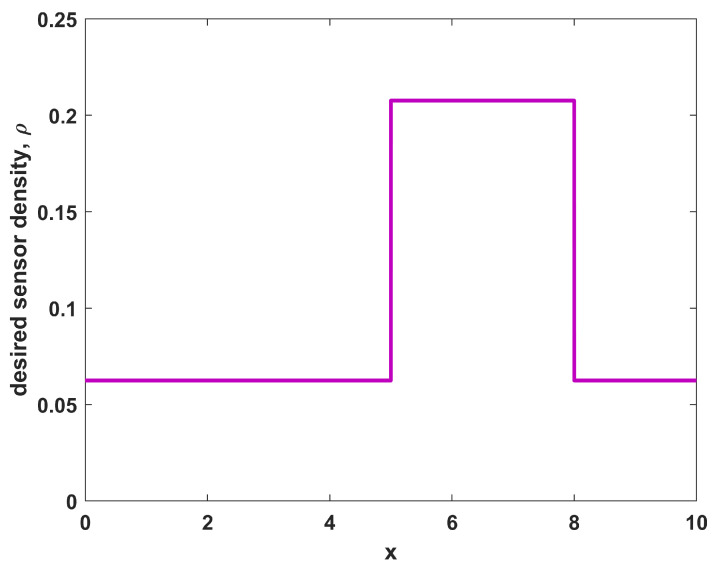
The sensor density for sensors in a uniform environment with desired non-uniform coverage.

Thus, there is a desire for larger coverage in a region in the middle (where 5≤x≤8) with a lesser need for coverage outside of that region, constituting a situation with a higher priority region near the middle of the domain. For this example, let us further consider the environmental characteristics in the region to be homogeneous, with r(x)=1∀x and $p_{d}\left(x\right)=0.5\;\forall x$. Thus, any sensor placed in the domain will observe the object of interest with probability 0.5 if the object is located within one unit of the sensor position. Applying these conditions to Equation (10) yields a sensor density function ρ(x) as shown in Figure 1. The desired sensor density ρ(x) for this case follows the shape of the desired coverage, which is expected for a uniform environment. While the shapes of ϕ(x) and ρ(x) are the same in this case, the specific levels of ρ(x) differ from those of ϕ(x) as they are based on not only the desired coverage ϕ(x), but also the number of available sensors $N^{TOT}$ and the sensor performance characteristics in the environment (r(x) and $p_{d}\left(x\right)$).

**Figure 2:**
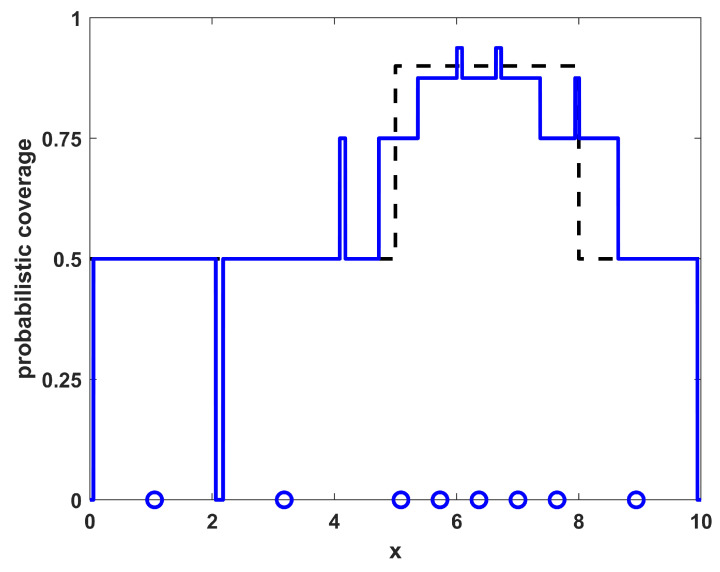
The resulting $N^{TOT}=8$ sampled sensor positions for example 1 (blue circles) along with their corresponding coverage (blue line). For reference, the desired coverage ϕ(x) is shown as a dashed black line.

Running a sampling procedure for $N^{TOT}=8$ sensors with the density ρ(x) as shown in Figure 1 leads to the sensor positions shown in Figure 2, where the specific resulting sensor locations are given by the circles along the x-axis. Also shown in Figure 2 is the resulting probabilistic coverage from the sensors, as given by $\eta (x;\left\{x_{i}\right\})$ in Equation (2), as well as the desired coverage ϕ(x) (for comparison). Note that the coverage obtained through sampling has a similar shape to the desired coverage; although it is generally larger because there are more sensors than required to achieve the desired coverage. The sampling procedure helps to maintain the shape in such situations. The quality of the match is clearly limited by the physical constraints on the environment and number of sensors available.

**Figure 3:**
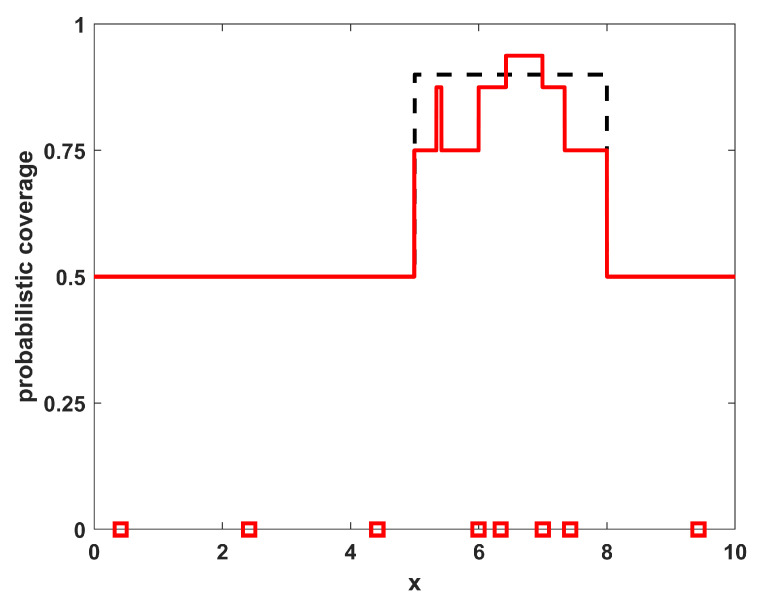
The resulting $N^{TOT}=8$ optimized sensor positions for example 1 (red squares) along with their corresponding coverage (red line). For reference, the desired coverage ϕ(x) is shown as a dashed black line.

To show the quality of the solutions obtained relative to the level of match that is achievable by this specific number of sensors with these specific environmental characteristics, a direct optimization of the coverage function for $\eta (x;\left\{x_{i}\right\})$ given in Equation (2) was also performed. As this function is made up of many segments of constant levels for $\eta_{i}\left(x\right)$, it is not differentiable and thus not amenable to gradient-based optimization approaches. The numerical optimization procedure utilized in the this paper employs a genetic algorithm metaheuristic, using a standard genetic algorithm [26] with single-point crossover, roulette selection, and an elitist selection strategy (maintaining the top two individuals from each generation). The parameters used were a population size of 50, a mutation probability of 1/64 (corresponding to one bit of mutation per individual in the population for each generation, on average), and each sensor’s x-location $x_{i}$ was represented with an 8 bit binary string. The algorithm was run for 1000 generations or to convergence if it converged earlier. The results of the optimized positions for this example are shown in Figure 3, where the sensor locations are shown along with the resulting coverage. Note that the locations and resulting coverage of the sampled solution in Figure 2 are similar to the optimized result in Figure 3. The benefit of the sampling approach over the numerical procedure is that the sampling approach is an analytic process that can provide solutions much more rapidly than the optimization approach, while still achieving many of the features from the optimal positioning of the sensors. In Table 1, the results of the sampling procedure are shown for the one-dimensional homogeneous environment with varying numbers of sensors $N^{TOT}$. The results shown are the quality of the match with the desired coverage function, specifically measured by the $L^{2}$ norm as $\int_{\Omega }{\left|\eta \left(x;\left\{x_{i}\right\}\right)-\phi \left(x\right)\right|}^{2}\;dx$. For comparison purposes, also included in the table are the quality of match that is optimally achievable for each number of sensors $N^{TOT}$. Note that both the sampled and optimized results show similar trends in that the match deteriorates for both very small and very large numbers of sensors, as expected.

**Figure 4:**
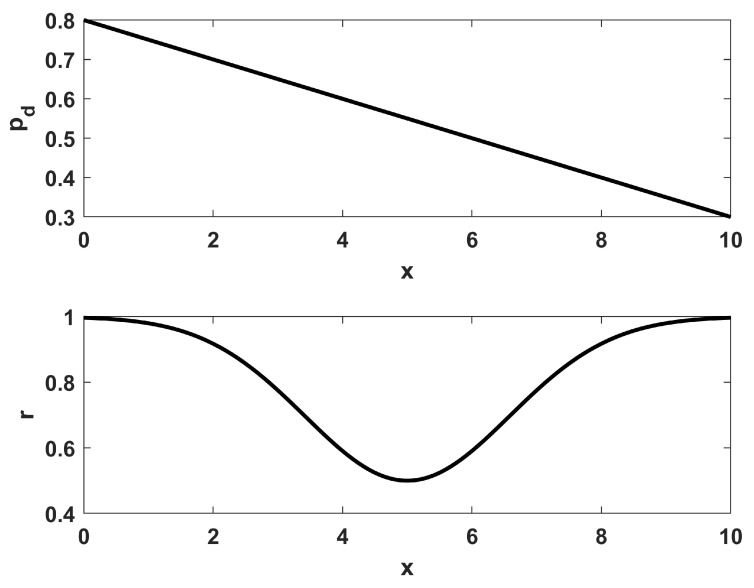
The environmentally varying spatial dependence for the probabilities $p_{d}\left(x\right)$ and sensor range r(x) for example 2.

**Figure 5:**
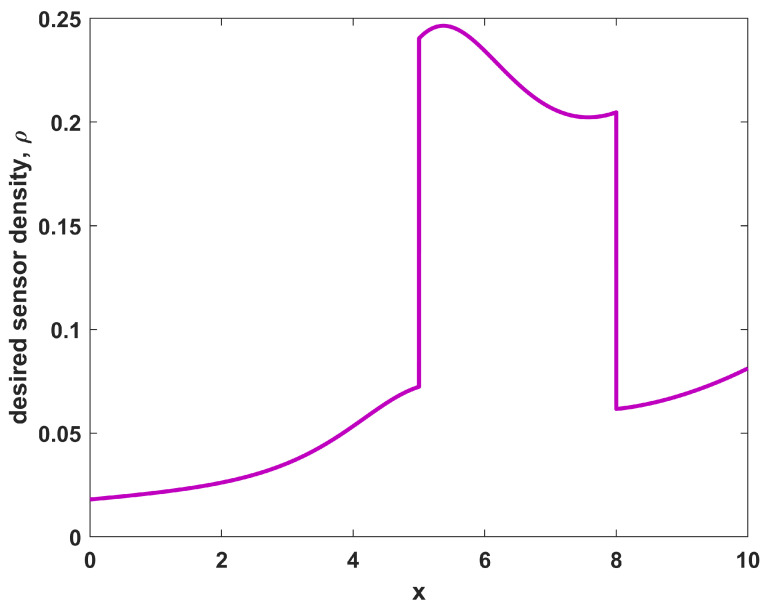
The sensor density ρ(x) corresponding to the non-homogeneous environment shown in Figure 4 and the desired non-uniform coverage of Equation (12).

Example 1 was a problem with uniform environmental characteristics, which is not practical. For a more realistic situation, example 2 considers the placement of $N^{TOT}=12$ sensors for the same desired coverage ϕ(x) that was given in Equation (12). However, in example 2 the environment is non-homogeneous, leading to a spatial dependency for both the sensor ranges r(x) as well as their probabilistic performance $p_{d}\left(x\right)$. These dependencies are shown in Figure 4, and the resulting desired sensor density function ρ(x) from Equation (10) is shown in Figure 5. Comparing Figure 5 to Figure 1 shows how the environmental effects have a great impact on the desired sensor density. In particular, more sensors are desired at the right side (near x=10) than the left side (near x=0) since the detection performance $p_{d}$ for individual sensors is lower there, requiring more overlap of coverage to achieve the goal. Also, there is a “bump” in the middle of the desired coverage region (from 5≤x≤8) that did not exist in Figure 1, owing to the fact that the environmental characteristics can have as great an effect on where to place sensors as the desired coverage trends.

**Figure 6:**
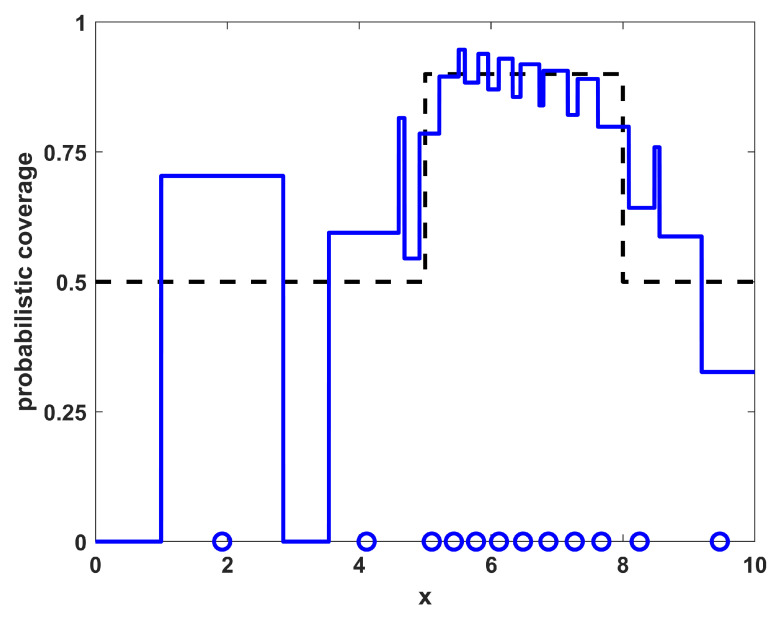
The resulting $N^{TOT}=12$ sampled sensor positions for example 2 (blue circles) along with their corresponding coverage (blue line). For reference, the desired coverage ϕ(x) is shown as a dashed black line.

**Figure 7:**
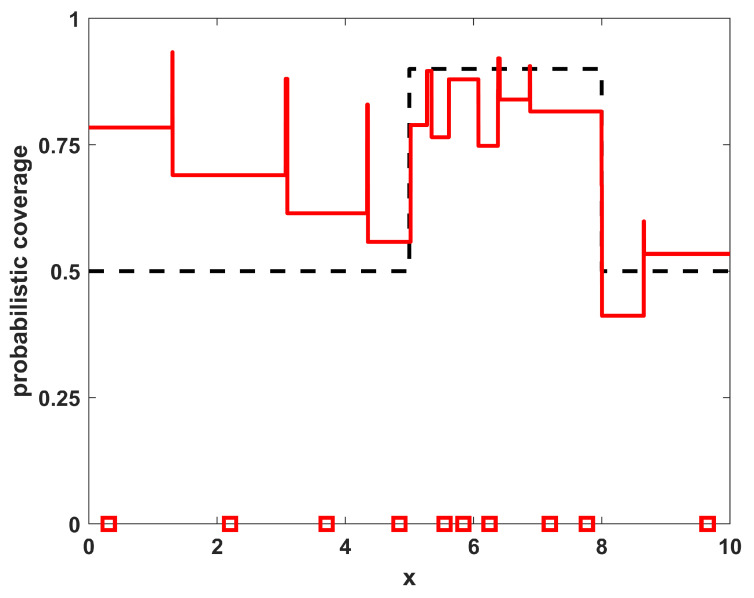
The resulting $N^{TOT}=12$ optimized sensor positions for example 2 (red squares) along with their corresponding coverage (red line). For reference, the desired coverage ϕ(x) is shown as a dashed black line.

Figure 6 and Figure 7 show the resulting placements of $N^{TOT}=12$ sensors for example 2 that were obtained using the analytic sampling approach and the optimization approach, respectively. As in the case with environmental homogeneity, for this case the sensor locations and the resulting coverage performance are qualitatively similar. The major difference between them is a sensor to the far left (near x=0.2) in the optimized result that is not in the sampled result. This is because the relatively small number of sensors makes the sampling approach somewhat inefficient in portions of the domain with low sensor density (small ρ(x)). However, that limited coverage is always going to be in a portion of the domain with lower coverage. The next example shows that this effect is not as prominent as the number of sensors increases.

**Figure 8:**
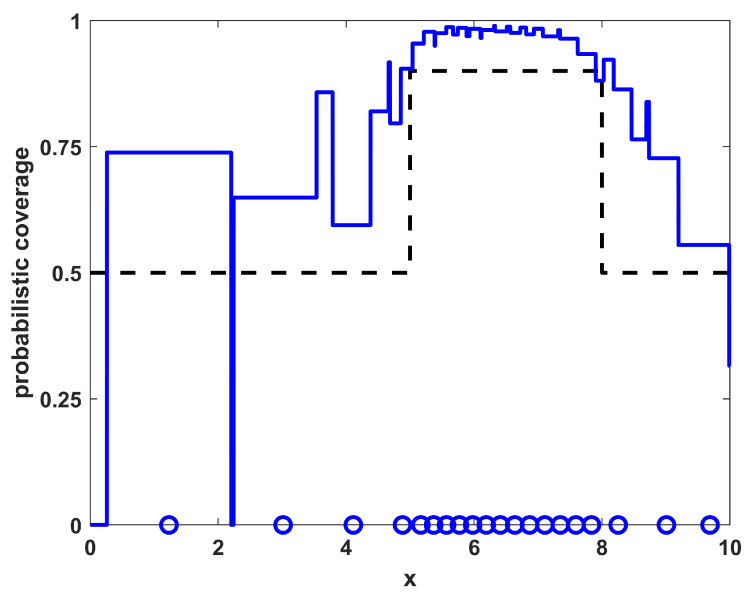
The resulting $N^{TOT}=20$ sampled sensor positions for example 3 (blue circles) along with their corresponding coverage (blue line). For reference, the desired coverage ϕ(x) is shown as a dashed black line.

**Figure 9:**
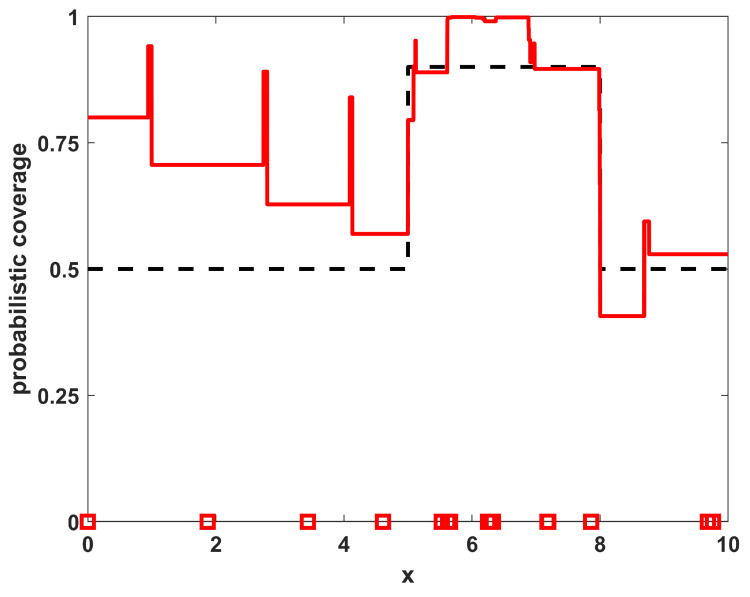
The resulting $N^{TOT}=20$ optimized sensor positions for example 3 (red squares) along with their corresponding coverage (red line). For reference, the desired coverage ϕ(x) is shown as a dashed black line.

For a third example, consider the same r(x) and $p_{d}\left(x\right)$ of example 2 (as shown in Figure 4) as well as the same desired coverage ϕ(x) from Equation (12). However, now $N^{TOT}=20$ sensors are placed in the region. In this dense sampling regime, the shape of the desired sensor density function ρ(x) is identical to that shown in Figure 5 for example 2, it only differs by a scaling factor of 20/12≈1.67 due to the $N^{TOT}$ term in Equation (10). The resulting sampled placements and the optimized placements for example 3 are shown in Figure 8 and Figure 9, respectively. From this example, it is shown that the large number of sensors leads to coverage well above the desired coverage levels. The sampling approach provides a scaled version of the sampling from example 2, packing more sensors into the area of higher desired coverage and spreading out the remainder accordingly. However, the optimization approach now tries to directly match the levels of the desired coverage, leading to lower coverage in some portions of the high-coverage region (from 5≤x≤8). In this sense, the sampling approach, in addition to being computationally much quicker than the optimization approach, may also provide solutions that are more desirable to the user (while not necessarily optimal in the $L^{2}$ sense). Table 2 shows the quality of the match of the results of the sampling procedure for the one-dimensional non-homogeneous environment with varying numbers of sensors $N^{TOT}$. The results are qualitatively similar to those seen in the homogeneous case in that the resulting match becomes worse for both very small and very large numbers of sensors $N^{TOT}$.

### 3.2. Example: Sensing in the Plane

**Figure 10:**
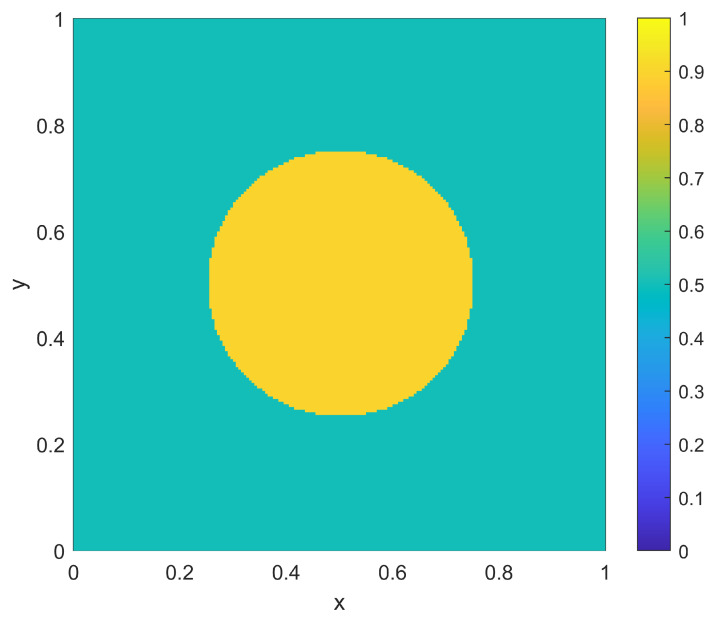
Coverage goal ϕ(x) for the two-dimensional examples.

For two-dimensional sensing, consider the situation where sensors are to be placed within a closed region $\Omega \subset R^{2}$. Specifically, take Ω to be the square region [0,1]×[0,1] for these examples. Such a domain may represent an area that is to be monitored or measured for some unusual activity or concentration. The sensors under consideration are described by a range r(x) and a probability $p_{d}\left(x\right)$ such that a sensor located at $x_{i}\in \Omega$ will cover the disc of radius $r(x_{i})$ that is centered at $x_{i}$ with a probability $p_{d}\left(x_{i}\right)$. For any practical application in such a domain, for sensor placement purposes the only concern is that the sensors are to be placed in Ω to best achieve some pre-defined desired level of probabilistic coverage ϕ(x). For the examples that follow, the two-dimensional coverage goal is defined as follows:

(13) $$\begin{array}{c} \phi \left(x\right)=\left\{\begin{array}{cc} 0.9, & |x-(0.5,0.5)|\leq 0.25\\ 0.5, & |x-(0.5,0.5)|>0.25 \end{array}\right. \end{array}$$

as shown in Figure 10. Note that this case has a desired nominal coverage level of 0.5 throughout most of the domain Ω, with a larger coverage level of 0.9 in a disc around the center, corresponding to a region of larger importance.

**Figure 11:**
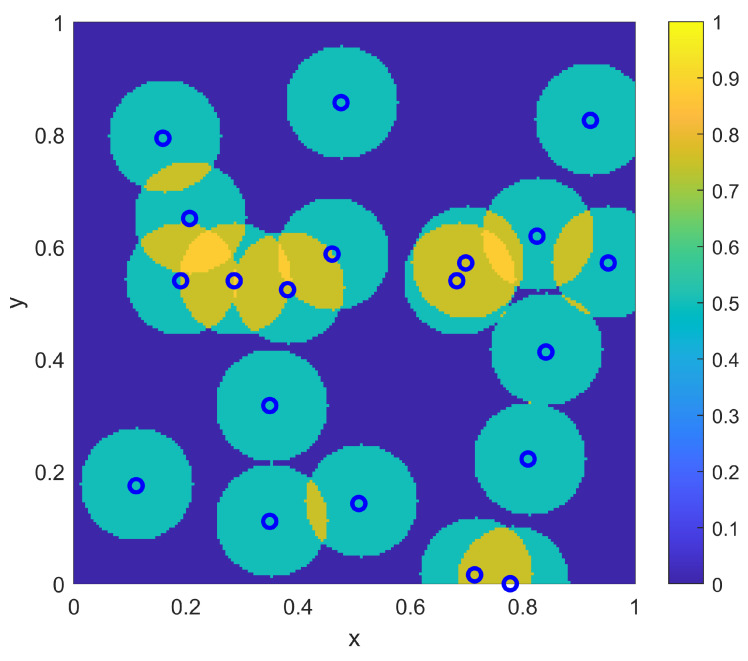
The resulting $N^{TOT}=20$ sampling-based sensor positions $\left\{x_{i}\right\}$ for the two-dimensional example with a uniform range of r(x)=0.1. Also shown is the resulting coverage $\eta (x;\left\{x_{i}\right\})$.

As a first example in this two-dimensional situation, consider a scenario where the environment is homogeneous with r(x)=0.1∀x and $p_{d}\left(x\right)=0.5\;\forall x$. For this homogeneous example, the goal is to determine the best locations $\left\{x_{i}\right\}$ to place $N^{TOT}=20$ sensors to provide a probabilistic coverage $\eta (x;\left\{x_{i}\right\})$ to best match the goal coverage ϕ(x). Note that a coverage range of r(x)=0.1 implies an individual sensor coverage of area of 0.0314, which is 1/32 of the total area of Ω. Thus, with $N^{TOT}=20$ total such sensors, there is not even an opportunity to cover the entire domain to the lower goal coverage level of 0.5. The question for sensor placement is to determine how much focus to put on overlapping sensors in the middle of Ω to achieve the desired higher coverage there versus spreading out sensors in the remainder of the domain to achieve the desired lower coverage there. Both the sampling-based placement and the optimization placement strategy provide this determination as part of their solutions. For the sampling approach, the sensor density ρ(x) of Equation (10) is computed to form the sampling distribution f(x) as in Equation (11). This associated CDF for the PDF f(x) is computed and then sampled deterministically to find the sensor locations $\left\{x_{i}\right\}$. The resulting sampled sensor locations for this example are shown in Figure 11, where the corresponding resulting coverage $\eta (x;\left\{x_{i}\right\})$ (as computed from Equation (2)) is also shown. From the resulting coverage, it is clear that the sampling approach provides a balance between spreading out some sensors to achieve the lower desired uniform coverage of 0.5 outside of the center of Ω while allowing some amount of overlap to achieve some of the larger desired coverage in the center of Ω.

**Figure 12:**
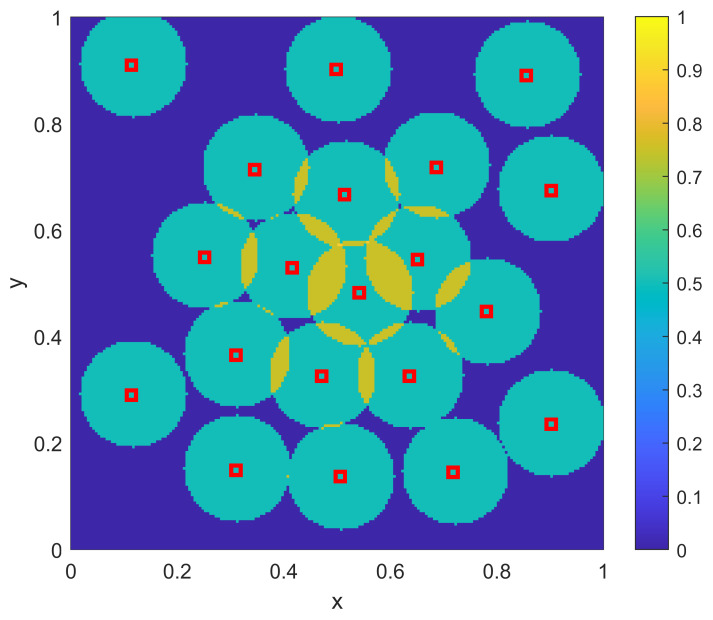
The resulting $N^{TOT}=20$ optimized sensor positions $\left\{x_{i}\right\}$ for the two-dimensional example with a uniform range of r(x)=0.1. Also shown is the resulting coverage $\eta (x;\left\{x_{i}\right\})$.

The optimization approach for this homogeneous two-dimensional example uses the same genetic algorithm approach that was used for the one-dimensional example, where the objective function is given as in Equation (3). In particular, the parameters $\left\{x_{i}\right\}$ are each represented by a 16-bit binary string (8 bits for each of the two dimensions). The standard genetic algorithm [26] is again utilized with single-point crossover, roulette selection and an elitist selection strategy. The other parameters used were a population size of 50 and a mutation rate of 1/320 (corresponding to one bit of mutation per individual in the population for each generation, on average). As in the one-dimensional examples, the genetic algorithm was run for 1000 generations, or to convergence if it converged earlier. The results of the optimization approach for the homogeneous two-dimensional example are shown in Figure 12. As in the sampling-based result of Figure 11, the optimization solution creates a balance between spreading out some sensors away from the center of Ω while allowing other sensors to provide the desired overlap in the center of Ω. Qualitatively, the relative split between these aspects is similar between the optimization and sampling-based approaches, and thus they provide similar levels of approximation to the desired coverage goal ϕ(x) that was shown in Figure 10. Table 3 shows the results of the $L^{2}$ quality of match from the coverage of the sampled sensors to the coverage goal ϕ(x) for varying numbers of sensors $N^{TOT}$. As in the one-dimensional case, the qualitative behavior of the match for the sampling approach is similar to that of the numerically intensive optimization approach.

**Figure 13:**
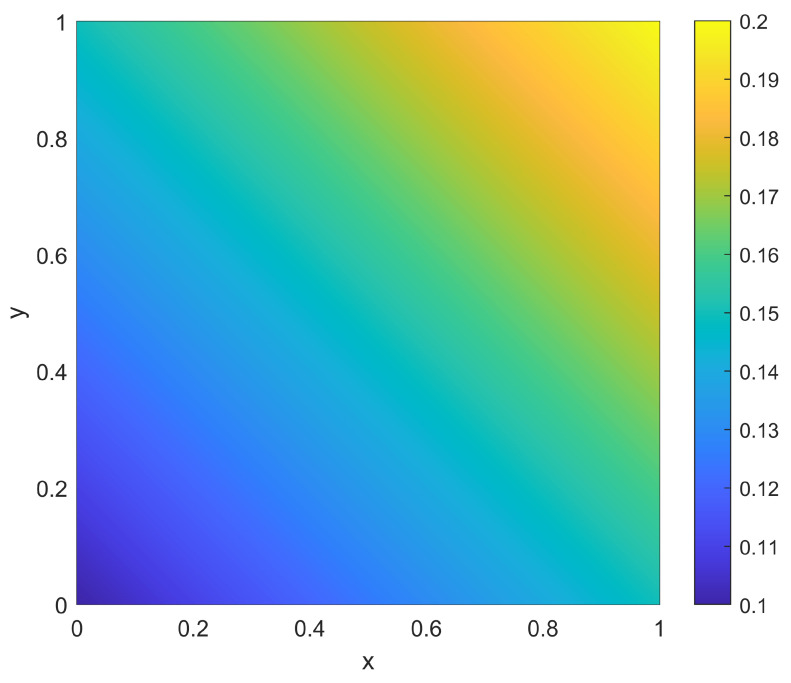
Spatial distribution of range r(x) for the two-dimensional example with a non-homogeneous environment.

As a second example of the two-dimensional situation, consider a scenario with a non-homogeneous environment in which the goal is to achieve the same coverage as shown in Figure 10. In this scenario, an individual sensor located at $x_{i}$ has a range $r(x_{i})$ that depends explicitly on its position, yet the probability $p_{d}\left(x\right)$ remains constant at $p_{d}\left(x\right)=0.5\;\forall x$. This is common when the sensing modality has physical properties that are heavily dependent on local environmental conditions. Consider the variation in range to be given by the function r(x) shown in Figure 13. This particular function was generated by taking the four values of r(x) at the corners of Ω to be {0.1,0.15,0.2,0.15} and performing a two-dimensional linear interpolation to obtain the values throughout Ω. For this non-homogeneous scenario, the goal of the placement procedure is to find the positions for placing $N^{TOT}=30$ sensors in the domain Ω.

**Figure 14:**
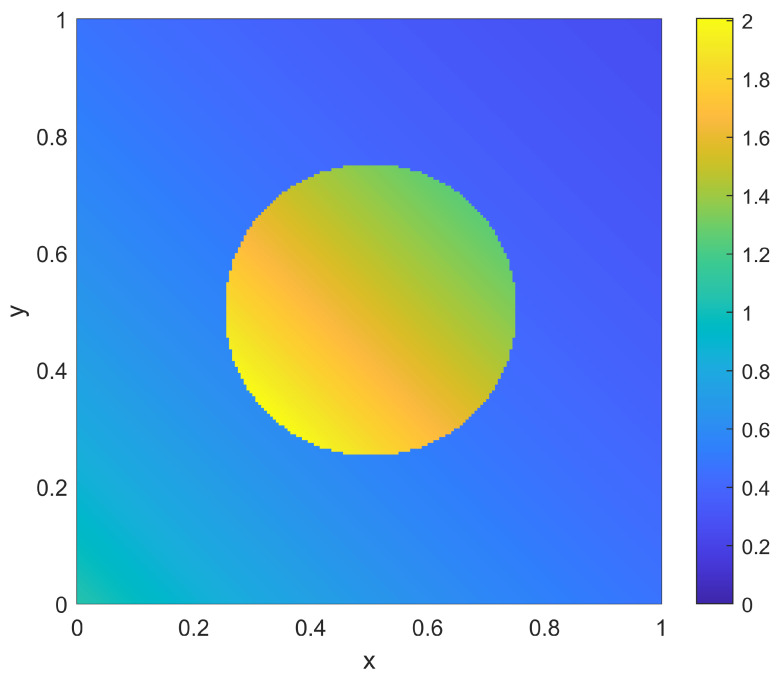
The sensor density ρ(x) for sensors in the two-dimensional non-uniform environment shown in Figure 13 along with goal non-uniform coverage ϕ(x) of Figure 10.

**Figure 15:**
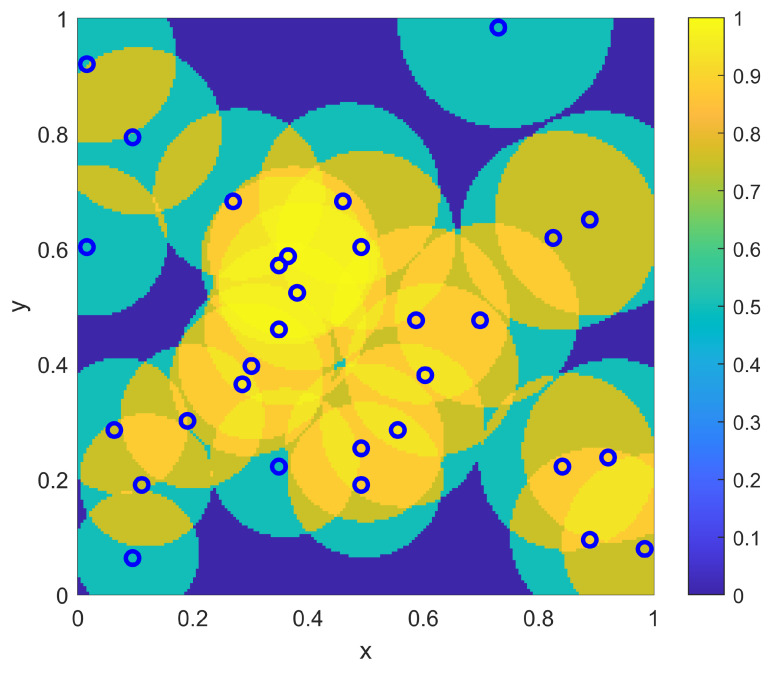
The resulting $N^{TOT}=30$ sampling-based sensor positions $\left\{x_{i}\right\}$ for the two-dimensional example with a nonuniform range of r(x) as shown in Figure 13. Also shown is the resulting coverage $\eta (x;\left\{x_{i}\right\})$.

For the sampling procedure, the sensor density ρ(x) from Equation (10) is no longer a simple scaling of the desired coverage ϕ(x), but instead is given as shown in Figure 14. Here there is still a desire to place more overlapping sensors in the center to achieve the higher desired coverage there, but there is now a trend to place more sensors to the bottom left (of both the disc in the center as well as the overall domain) in order to compensate for the range variations as per Figure 13. The sample-based resulting positions $\left\{x_{i}\right\}$ and coverage $\eta (x;\left\{x_{i}\right\})$ are shown in Figure 15. As in the homogeneous case, it is clear that the sampling approach again provides a balance between spreading out sensors across Ω to achieve the lower coverage while allowing some overlap near the center of Ω to achieve the larger desired coverage there. However, as opposed to the homogeneous case, there are more sensors near the bottom left to account for the lower sensor ranges r(x) found there.

**Figure 16:**
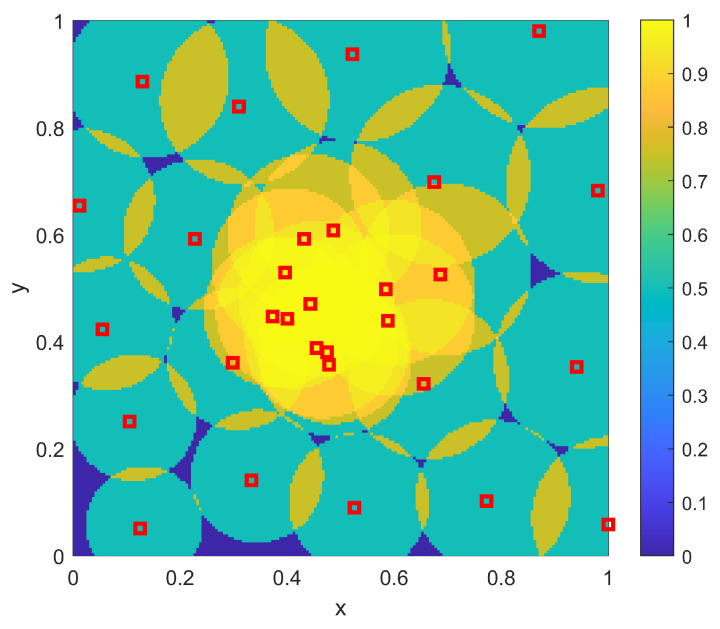
The resulting $N^{TOT}=30$ optimized sensor positions $\left\{x_{i}\right\}$ for the two-dimensional example with a nonuniform range of r(x) as shown in Figure 13. Also shown is the resulting coverage $\eta (x;\left\{x_{i}\right\})$.

For the optimization procedure for the non-homogeneous two-dimensional example, the same genetic algorithm procedure was used as for the homogeneous example (including the same parameter settings). The resulting optimal sensor placements $\left\{x_{i}\right\}$ and the corresponding coverage $\eta (x;\left\{x_{i}\right\})$ are shown in Figure 16. This optimized example provides a very close match to the desired coverage ϕ(x) of Figure 10. Note that this solution has the same qualitative features of the sampling-based approach, in that the balance between overlapping sensors in the middle and those spread out around the remainder of the domain Ω is similar to that seen in the sampling-based approach. Also, both approaches are affected similarly by the range variation that was shown in Figure 13. While the optimization is clearly a better result, what is important here is that the sampling-based approach provides much of the qualitative features of the optimal solution, without the need to run an optimization algorithm. Thus, a rapidly computed analytical solution to finding where to position sensors has been obtained that provides performance that is close to the performance of a large-scale computational approach. Table 4 shows the results of the $L^{2}$ quality of match from the coverage of the sampled sensors to the coverage goal ϕ(x) for varying numbers of sensors $N^{TOT}$. The results are similar to those seen in the homogeneous case.

## 4. Conclusions

A sampling-based approach has been developed for determining the positioning of distributed sensors to achieve a desired cooperative coverage goal. This approach is completely analytic, and thus appropriate for low-computation solutions, such as for repositioning sensors in the field, or when making sensor positioning decisions onboard an autonomous deployment platform. Numerical examples have been presented to show the efficacy of the approach in both one-dimensional and two-dimensional settings, and the numerical results were compared against optimal solutions that were computed using a large-scale metaheuristic numerical optimization procedure. In all cases, the sampling-based approach provided qualitatively similar results to the optimization procedure, thus validating the utility of this sampling-based approach as a method for rapidly determining a good set of sensor positions to obtain coverage that approaches the pattern desired for a coverage goal. Future extensions of this work include the use of improved numerical methods for the sampling of CDFs in higher dimensions as well as the development of practical techniques for deriving desired coverage functions ϕ(x) from multiple design goals.

## Figures and Tables

**Table 1:** Placement results in a one-dimensional homogeneous environment.

$N^{TOT}$	$L^{2}$ for Sampling	$L^{2}$ for Optimization
4	0.3380	0.3129
8	0.1207	0.0626
12	0.1606	0.0396
16	0.2242	0.0461
20	0.2714	0.0931
30	0.3467	0.1674

**Table 2:** Placement results in a one-dimensional non-homogeneous environment.

$N^{TOT}$	$L^{2}$ for Sampling	$L^{2}$ for Optimization
4	0.4262	0.3987
8	0.2963	0.2116
12	0.2416	0.1479
16	0.2180	0.1415
20	0.2131	0.1458
30	0.2786	0.1472

**Table 3:** Placement results in a two-dimensional homogeneous environment.

$N^{TOT}$	$L^{2}$ for Sampling	$L^{2}$ for Optimization
20	0.4343	0.3666
30	0.3696	0.2768
40	0.3375	0.2053
60	0.3002	0.1535
80	0.2846	0.1622
100	0.2795	0.1938

**Table 4:** Placement results in a two-dimensional non-homogeneous environment.

$N^{TOT}$	$L^{2}$ for Sampling	$L^{2}$ for Optimization
20	0.3139	0.1667
30	0.3307	0.1384
40	0.3196	0.1335
60	0.3348	0.1672
80	0.3613	0.2236
100	0.3852	0.2676

## Data Availability

No new data were created or analyzed in this study. Data sharing is not applicable to this article.

## References

[1] Qi H., Iyengar S., Chakrabarty K. (2001). Distributed sensor networks—A review of recent research. J. Frankl. Inst..

[2] Akyildiz I., Su W., Sankarasubramaniam Y., Cayirci E. (2002). A survey on sensor networks. IEEE Commun. Mag..

[3] Wang B. (2011). Coverage problems in sensor networks: A survey. ACM Comput. Surv..

[4] Farahani R.Z., Asgari N., Haidari N., Hosseininia M., Goh M. (2012). Covering problems in facility location: A review. Comput. Ind. Eng..

[5] Berman O., Drezner Z., Krass D. (2009). Cooperative cover location problems: The planar case. IIE Trans..

[6] Berman O., Drezner Z., Krass D. (2011). Discrete cooperative covering problems. J. Oper. Res. Soc..

[7] Farsi M., Elhosseini M.A., Badawy M., Ali H.A., Eldin H.Z. (2019). Deployment techniques in wireless sensor networks, coverage and connectivity: A survey. IEEE Access.

[8] Gu Y., Ji Y., Li J., Zhao B. (2009). QoS-aware target coverage in wireless sensor networks. Wirel. Commun. Mob. Comput..

[9] Wettergren T.A., Costa R. (2021). Optimal configuration planning for sensor network serviceability under a system coverage constraint. IEEE Access.

[10] Deif D.S., Gadallah Y. (2014). Classification of wireless sensor network deployment techniques. IEEE Commun. Surv. Tutor..

[11] Lin F.Y.S., Chiu P.L. (2005). A near-optimal sensor placement algorithm to achieve complete coverage/discrimination in sensor networks. IEEE Commun. Lett..

[12] Singh A., Rossi A., Sevaux M. (2013). Matheuristic approaches for Q-coverage problem versions in wireless sensor networks. Eng. Optim..

[13] Wu Q., Rao N.S.V., Du X., Iyengar S.S., Vaishnavi V.K. (2007). On efficient deployment of sensors on planar grid. Comput. Commun..

[14] Liu X. (2015). A deployment strategy for multiple types of requirements in wireless sensor networks. IEEE Trans. Cybern..

[15] Heo N., Varshney P.K. (2005). Energy-efficient deployment of intelligent mobile sensor networks. IEEE Trans. Syst. Man Cybern. Part A Syst. Hum..

[16] Jaramillo J.H., Bhadury J., Batta R. (2002). On the use of genetic algorithms to solve location problems. Comput. Oper. Res..

[17] Yoon Y., Kim Y.H. (2013). An efficient genetic algorithm for maximum coverage deployment in wireless sensor networks. IEEE Trans. Cybern..

[18] Wettergren T.A., Costa R. (2009). Optimal placement of distributed sensors against moving targets. ACM Trans. Sens. Netw..

[19] Kumar S., Lai T.H., Arora A. (2007). Barrier coverage with wireless sensors. Wirel. Netw..

[20] Saipulla A., Westphal C., Liu B., Wang J. (2013). Barrier coverage with line-based deployed mobile sensors. Ad Hoc Netw..

[21] Si P., Ma J., Tao F., Fu Z., Shu L. (2020). Energy-efficient barrier coverage with probabilistic sensors in wireless sensor networks. IEEE Sens. J..

[22] Bar-Noy A., Baumer B., Rawitz D. (2017). Set it and forget it: Approximating the set once strip cover problem. Algorithmica.

[23] Bhattacharya B., Burmester M., Hu Y., Kranakis E., Shi Q., Wiese A. (2009). Optimal movement of mobile sensors for barrier coverage of a planar region. Theor. Comput. Sci..

[24] Leonard N.E., Olshevsky A. (2013). Nonuniform coverage control on the line. IEEE Trans. Autom. Control.

[25] Watfa M.K., Commuri S. (2009). Self organization of sensor networks for energy-efficient border coverage. J. Commun. Netw..

[26] Goldberg D.E. (1989). Genetic Algorithms in Search, Optimization, and Machine Learning.

